# The potential of routine surveillance data for identifying the needs of people living with HIV among migrants: Description of German HIV notifications in the context of the Ukrainian refugee reception, 2022–2023

**DOI:** 10.1186/s12889-026-26787-6

**Published:** 2026-02-28

**Authors:** Klara Röbl, Martin Friebe, Christian Kollan, Ulrich Marcus, Barbara Gunsenheimer-Bartmeyer

**Affiliations:** 1 Department of Infectious Disease Epidemiology, Robert Koch-Institute, Berlin, Germany; 2 Postgraduate Training for Applied Epidemiology (PAE), Robert Koch-Institute, Berlin, Germany; 3 ECDC Fellowship Programme, Field Epidemiology path (EPIET), European Centre for Disease Prevention and Control (ECDC), Stockholm, Sweden

**Keywords:** HIV, Ukraine, Germany, Policy

## Abstract

**Background:**

Since the start of Russia’s war against Ukraine, more than one million people from Ukraine – a country with high Human Immunodeficiency Virus (HIV)-prevalence – have fled to Germany. We aimed to estimate the number of people living with HIV (PLHIV) with a link to Ukraine reported to the German HIV notification system, analyse their characteristics and estimate the proportion of prior known infections to better understand the care needs of this population.

**Methods:**

We descriptively analysed HIV surveillance data from 2001 to 2023 regarding links to Ukraine. We further characterised HIV notifications received between March 2022 and December 2023 with either a link to Ukraine or Germany in terms of reported transmission routes, age and gender distributions. We estimated the proportions of prior known and probably prior known diagnoses based on notifier comments, reported CD4 cell count and viral load measurements.

**Results:**

We found a steep increase in Ukraine-linked notifications starting in March 2022, with yearly proportions reaching 19.7% in 2022 and 13.1% in 2023, compared to only 0.2%-1.6% in previous years. Among PLHIV from Ukraine, most were female (60%), and the majority of adult cases reported heterosexual transmission (74%). In contrast, notifications linked to Germany predominantly involved men who have sex with men (MSM) (45%). Compared to notifications about PLHIV linked to Germany, those about PLHIV associated to Ukraine showed a higher proportion of children (5% vs. 2%) – the majority of whom were infected through pre- and perinatal transmission (89%). The majority of PLHIV from Ukraine had either prior known or probably prior known diagnoses (53% of adults, 75% of children).

**Conclusions:**

Thus, PLHIV from Ukraine differ in their characteristics from those linked to Germany and consequently in their needs regarding HIV prevention and care services. They require rapid access to healthcare structures in Germany to ensure continuity of treatment. Our findings highlight the importance of considering migrant and refugee populations within Germany’s national HIV strategies, which are currently focused primarily on MSM. While our results demonstrate the overall usefulness of HIV surveillance data for characterising migrant populations, they also call for improvements in data quality and completeness.

**Supplementary Information:**

The online version contains supplementary material available at 10.1186/s12889-026-26787-6.

## Background

In 2023, an estimated 40 million people worldwide were living with Human Immunodeficiency Virus (HIV) infections, including approximately 1.4 million children under 15 years of age [1]. In the same year, about 1.3 million new HIV infections were recorded globally, of which around 120,000 occurred in children [1]. Within the World Health Organization (WHO) European Region, these figures amounted to a total of about 3.1 million people living with HIV (PLHIV) and 160,000 new infections in 2023 [1]. The highest incidences were observed in the eastern part of the region, which includes Ukraine and accounted for approximately 72% of all new HIV infections in the WHO European region in 2022 [2]. With 29.8 new infections per 100,000 population in 2022, Ukraine reported the second-highest incidence within the region [2]. Estimates of prevalence in the general population in Ukraine range from 0.66% (2021; Ukrainian Ministry of Health) to 1.0% (2019; UNAIDS) [3, 4]. In contrast, Germany reported a prevalence of around 0.1% and approximately 2,200 new infections in 2021, corresponding to an incidence of about 2.7 per 100,000 population [5, 6].

Since the onset of Russia’s war against Ukraine in February 2022 and up to July 2024, approximately 1,183,700 people from Ukraine have sought refuge in Germany [7]. In 2022, Germany recorded an increase of roughly 1,000 newly reported HIV infections compared to 2021, of which around 700 were potentially concerning infections in newly arrived migrants from Ukraine that have been diagnosed before [8]. Currently, published figures for Germany only include notifications meeting the national reference definition of confirmed HIV cases, which requires either and antibody confirmation test (Western Blot) or nucleic acid detection exceeding 1,000 copies/ml. This definition might underestimate the number of PLHIV who migrated from Ukraine, as it excludes individuals receiving treatment with potentially lower viral loads.

Given the high prevalence of HIV in Ukraine and prior descriptions of PLHIV from Ukraine within European Union/European Economic Area (EU/EEA) countries [2], we hypothesize that a considerable proportion of HIV notifications about PLHIV from Ukraine who arrived in Germany since early 2022 concern previously diagnosed infections. These individuals require rapid treatment continuation in Germany. To design people-centred and effective public health strategies, evidence on the characteristics and needs of this population in terms of HIV prevention and care is essential [2, 9].

We therefore aimed to (1) estimate the number and proportion of HIV notifications in Germany about PLHIV who migrated from Ukraine based on routine national surveillance data, (2) describe their characteristics in comparison with PLHIV linked to Germany, and (3) determine the proportion of known and probably known infections to better understand this population’s care needs and inform HIV care policy and planning.

## Methods

### Data source and HIV notification process in Germany

According to § 7, Paragraph 3 of the German Infection Protection Act (IfSG), all detections of HIV using direct (e.g., virus cultivation or nucleic acid testing) or indirect methods (e.g., antibody detection) in Germany are notifiable to the Robert Koch Institute (RKI) – Germany’s national public health institute – under pseudonyms derived from the person’s name. To avoid duplicates, notifications are automatically compared with existing records based on the following criteria: pseudonyms, month and year of birth, gender, and regional classification [8]. In recent years, approximately 10% of all notifications have been identified as duplicates.

Although the HIV notification system in Germany is currently being digitalized, notifications were still received on paper during the study period [10]. Notifications forms are intended to include information on the country of origin, country of infection, gender, testing procedure, previous HIV diagnostics in Germany, clinical details, route of transmission and optional comments by the notifying person. The latter may contain additional information, such as prior HIV diagnoses made abroad [11]. However, these fields are not mandatory and are therefore incomplete for many notifications.

RKI’s annual published reports only include data on notifications that meet the reference definition. This definition includes notifications with either a positive antibody test or a viral load exceeding 1,000 copies/ml [8]. 

For this study, we analysed all HIV notifications received within the German routine surveillance system between 2001 and 2023, including those that did not meet the reference definition. We chose this approach because HIV notifications concerning PLHIV from Ukraine may not necessarily fulfil the reference criteria: diagnostics conducted for treatment continuation of previously known infections may involve only viral load assessments, which – according to WHO recommendations – are used for treatment monitoring and may yield values below the 1,000 copies/ml threshold [9]. Consequently, restricting analyses to notifications that meet the reference definition would likely underestimate the extent of PLHIV from Ukraine living in Germany.

### Definitions

#### Notifications fulfilling the reference definition

Notifications fulfilling the reference definition refer to reports of a first HIV detection in Germany confirmed by either antibody testing or nucleic acid detection with viral loads exceeding 1,000 copies/ml [8, 12]. 

Notifications not fulfilling the reference definition may include reports with viral loads below 1,000 copies/ml – or even below detection limit –, combinations of positive antibody tests with undetectable viral loads, or repeat notifications about PLHIV whose infection had already been reported in Germany.

However, fulfilling the reference definition does not necessarily imply a newly acquired HIV infection, as the definition is solely based on the first positive test conducted in Germany.

Notifications not fulfilling the reference definition may also include false positives – particularly among children –, if reports concern the detection of maternal antibodies transferred to infants. For clarity, we refer to notifications fulfilling the reference definition as ‘confirmed infections’.

#### Country links

We established country links to Ukraine and Germany based the reported country of origin. If this information was missing, the reported country of infection was used instead. In cases where the reported country of origin differed but the reported country of infection was either Germany or Ukraine, we assumed country links to the latter, as PLHIV with other nationalities might have migrated from Ukraine to Germany. Notifications with both another country of origin and another country of infection, or a combination of one non-German/non-Ukrainian country and missing information on the other variable, were categorized as ‘other’. For clarity, we refer to notifications regarding PLHIV linked to Ukraine or Germany as ‘PLHIV from UKR’ and ‘PLHIV from GER’, respectively.

#### PLHIV with prior known diagnoses

We categorized infections as known prior to notification, i.e., not newly diagnosed, when notifier comments explicitly mentioned a previous HIV diagnosis made abroad.

#### PLHIV with probably prior known diagnoses

Among PLHIV for whom no notifier comment indicated a prior diagnosis abroad, we used reported viral load and CD4 cell count data as proxies for infection stage to identify PLHIV with probably previously known diagnoses. We assumed that the HIV infection was probably already known and treated for those notifications with reported viral loads up to 200 copies/ml and/or reported CD4 cell counts above 500 cells/µl. Viral loads up to 200 copies/ml are considered indicative of viral suppression; while CD4 cell counts between 500 and 1,500/µl fall within the normal range; both parameters can indicate a successfully treated infection [13–15]. 

### Analysis

We performed descriptive analysis of yearly proportions of notifications according to the established country links from 2001 to 2023, and of monthly proportions from January 2022 onward. For notifications about PLHIV from GER and UKR as of March 2022, we conducted more detailed analyses, focusing on gender and age distribution, reported transmission routes, and the proportion of prior known or probably prior known diagnoses, including the extent of missing data, with a separate description of notifications about children under 15 years of age.

All analyses were conducted both for the whole dataset and stratified by confirmed infections, in order to identify differences between confirmed and non-confirmed notifications and to account for false positives.

A description of PLHIV from GER is presented to enable a comparison of characteristics of PLHIV without migration background.

All analyses were conducted using R, version 4.1.3 (R Foundation for Statistical Computing, Vienna, Austria).

### Data protection

Positive HIV diagnostics in Germany are notified using pseudonyms derived from the individual’s name. All data analysed in this study were pseudonymised and the authors had no access to identifiable personal information at any stage of the analysis.

## Results

### Notifications since January 2001

Between 2001 and 2023, a total of 102,336 HIV notifications were recorded in Germany. Of these, 1,897 (2%) were linked to Ukraine (UKR), 54,869 (54%) to Germany (GER) and 19,850 (19%) to other countries. For 25,720 (25%) notifications, we were unable to establish a country link due to missing information on both the country of origin and the country of infection. Among a total of 65,245 (64%) notifications fulfilling the reference definition (confirmed infections), the proportion without a country link was considerably lower (*n* = 3,861, 6%), while the proportions of PLHIV from UKR (*n* = 1,680, 3%) and GER (*n* = 44,583, 68%) were higher.

**Fig. 1:**
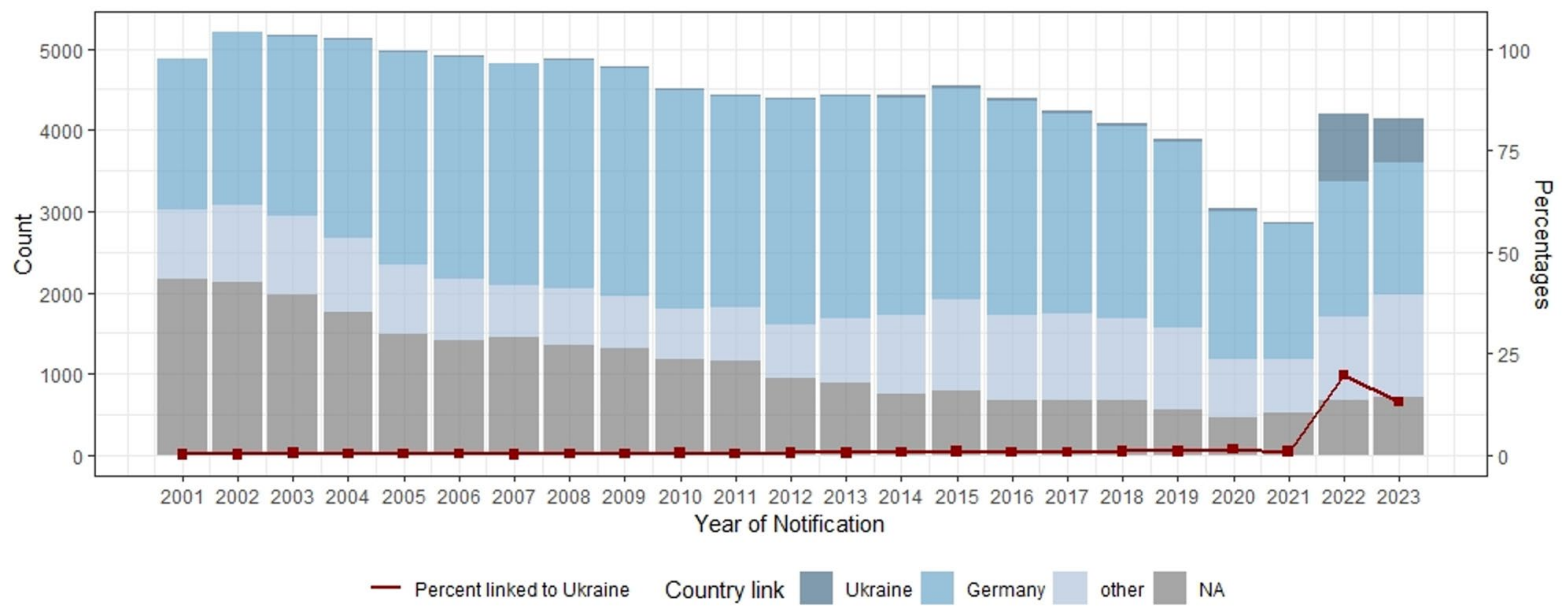
Yearly HIV-notification counts by country link (including unconfirmed infections), 2001–2023

When comparing across the years, the proportion of PLHIV from UKR increased in the years 2022 and 2023, reaching 19.7% and 13.1%, respectively, – compared to only 0.2% to 1.6% in the years 2001–2021. Among confirmed infections, these proportions were even higher – 22.5% in 2022 and 15.7% in 2023. Figure 1 presents yearly notification counts by country link for all notifications, along with the yearly proportions of PLHIV from UKR.

### Notifications since March 2022

Between March 2022 and December 2023, a total of 7,850 HIV notifications were recorded in Germany. Of these, 1,362 (17%) concerned PLHIV from UKR and 3,049 (39%) PLHIV from GER. Among a total of 6,228 (79%) confirmed infections, the proportions of PLHIV from UKR (*n* = 1,255, 20%) and from GER (*n* = 2,662, 43%) were both higher compared to the overall dataset.

**Fig. 2:**
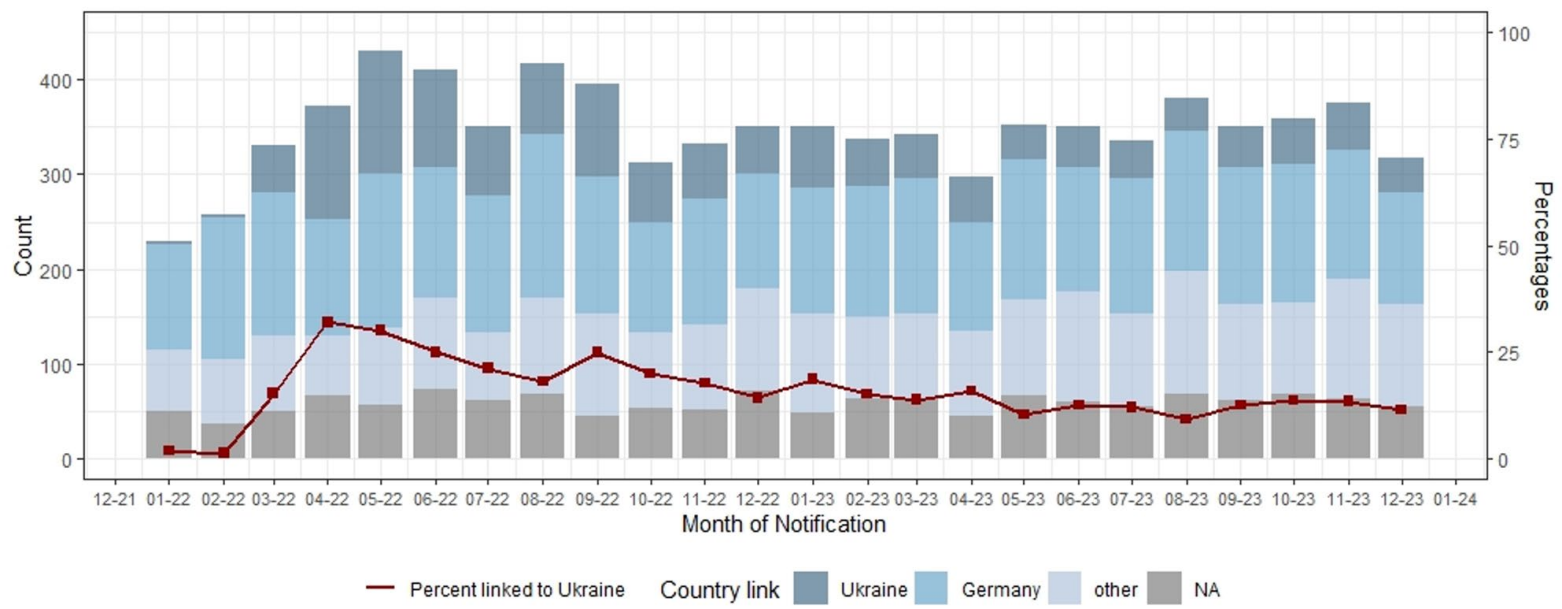
Monthly HIV-notification counts by country link (including unconfirmed infections), 01/2022-12/2023

The monthly proportion of PLHIV from UKR peaked in April 2022, reaching 32% (*n* = 119) among all notifications and 38% (*n* = 104) among confirmed infections. Thereafter, the proportion declined but remained consistently above pre-war levels until the end of the study period in December 2023, with a mean monthly proportion of 17% (mean *n* = 62) among all notifications and 20% (mean *n* = 57) among confirmed infections. Figure 2 displays monthly notification counts by country link for all notifications, along with the corresponding proportions of PLHIV from UKR.

### Characteristics of PLHIV, March 2022 – December 2023

Between March 2022 and December 2023, 92% (*n* = 1,255) of notifications about PLHIV from UKR and 87% (*n* = 2,662) of those concerning PLHIV from GER were confirmed infections. The mean reported age among adults was comparable between both groups – 41.1 years for PLHIV from UKR and 43.2 years for PLHIV from GER. The proportion of children (< 15 years) living with HIV was higher among PLHIV from UKR (*n* = 101; 7%) than among those from GER (*n* = 60;2%) (Table 1).

**Table 1:** Characteristics of PLHIV from UKR and GER, 03/2022-12/2023

		UKR *N* = 1,362	GER *N* = 3,049
Reference definition	Fulfilled	1,255 (92%)	2,662 (87%)
	Not fulfilled	106 (8%)	398 (13%)
Gender	Female	821 (60%)	488 (16%)
	Male	540 (40%)	2,559 (84%)
	Missing	1 (0%)	2 (0%)
Age	Mean age in years	38.8	42.3
	Adults (%; mean age)	1,286 (94%; 40.6)	2,972 (97%; 43.2)
	Children < 15 years (%; mean age)	74 (5%; 7.5)	58 (2%; 0.5)
	Missing	2 (0%)	19 (1%)
Transmission route adults > = 15 years	MSM	64 (5%)	1,335 (45%)
	Injecting drug use	245 (20%)	176 (6%)
	Haemophilia	0 (0%)	3 (0%)
	Blood transfusion/products	0 (0%)	1 (0%)
	Heterosexual	949 (74%)	309 (10%)
	Pre- or perinatal	26 (2%)	6 (0%)
	Missing	2 (0%)	1,142 (38%)
Transmission route children < 15 years	MSM	0 (0%)	0 (0%)
	Injecting drug use	0 (0%)	0 (0%)
	Haemophilia	0 (0%)	0 (0%)
	Blood transfusion/products	0 (0%)	0 (0%)
	Heterosexual	2 (3%)	1 (2%)
	Pre- or perinatal	66 (89%)	48 (83%)
	Missing	6 (8%)	9 (16%)
CD4 cell count	< 200/µl	124 (9%)	306 (10%)
	200–500/µl	164 (12%)	322 (11%)
	> 500/µl	359 (26%)	233 (8%)
	Missing	715 (53%)	2,188 (72%)
Viral load	Below detection limit	438 (32%)	94 (3%)
	Detection limit - <=200 copies/ml	46 (3%)	20 (1%)
	200–999 copies/ml	20 (2%)	47 (2%)
	>=1,000 copies/ml	285 (21%)	1,314 (43%)
	Missing	573 (42%)	1,574 (52%)
Prior known diagnosis	Notifier comment indicating diagnosis made abroad	499 (37%)	11 (0%)
	Missing comment	863 (63%)	3,038 (100%)

### Gender

**Fig. 3:**
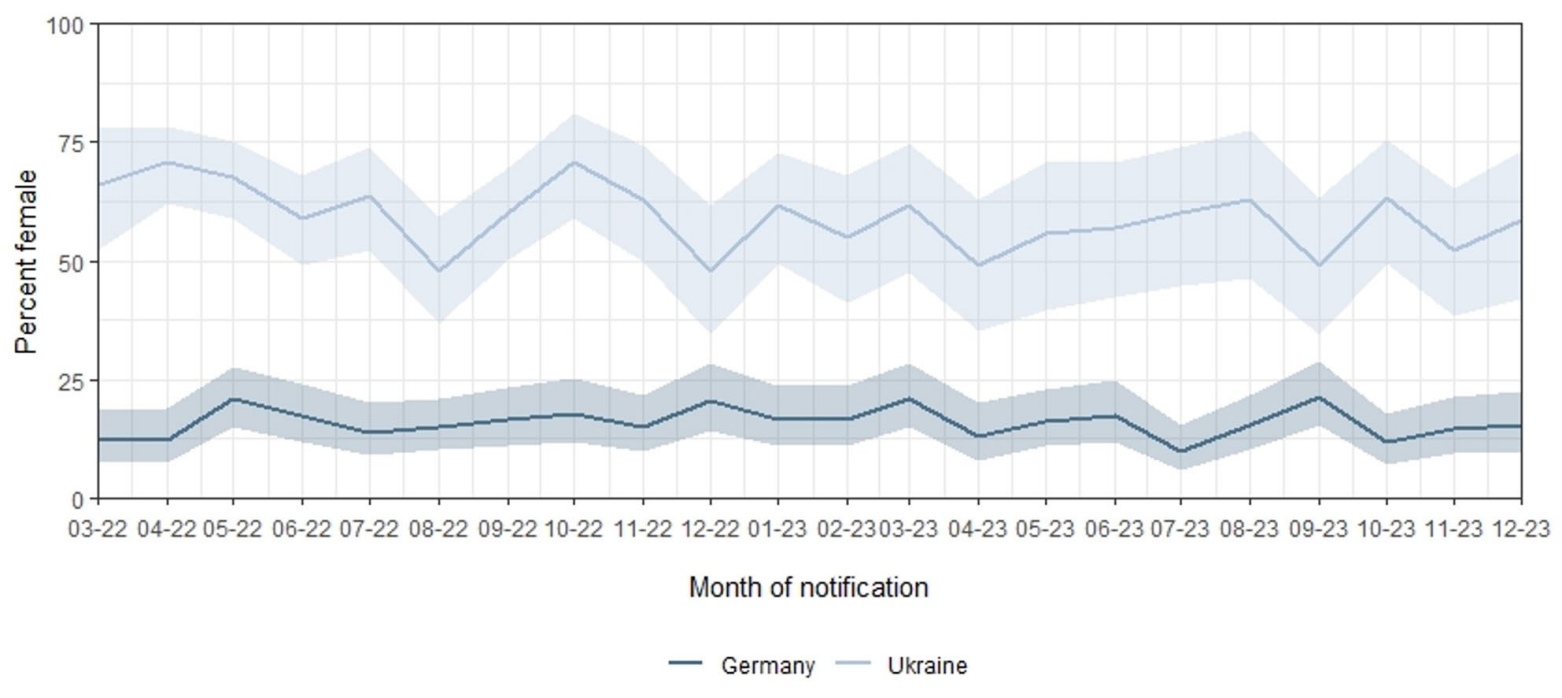
Monthly proportion and 95%-confidence interval of female PLHIV from UKR and GER Germany, 03/2022-12/2023

Between March 2022 and December 2023, the majority of PLHIV from UKR were female (*n* = 821; 60%), whereas those from GER were predominantly male (*n* = 2,559; 84%). (Table 1). The proportion of notifications about female PLHIV remained consistently higher among those from UKR throughout the entire study period, both for all notifications and for confirmed infections, ranging from 48% in August 2022 to 71% in April and October 2022. In contrast, among PLHIV in Germany, the proportion of females ranged from 10% in July 2023 to 22% in September 2023. Figure 3 shows monthly proportions of female PLHIV from UKR and GER among all notifications.

### Transmission routes

#### Adults

**Fig. 4:**
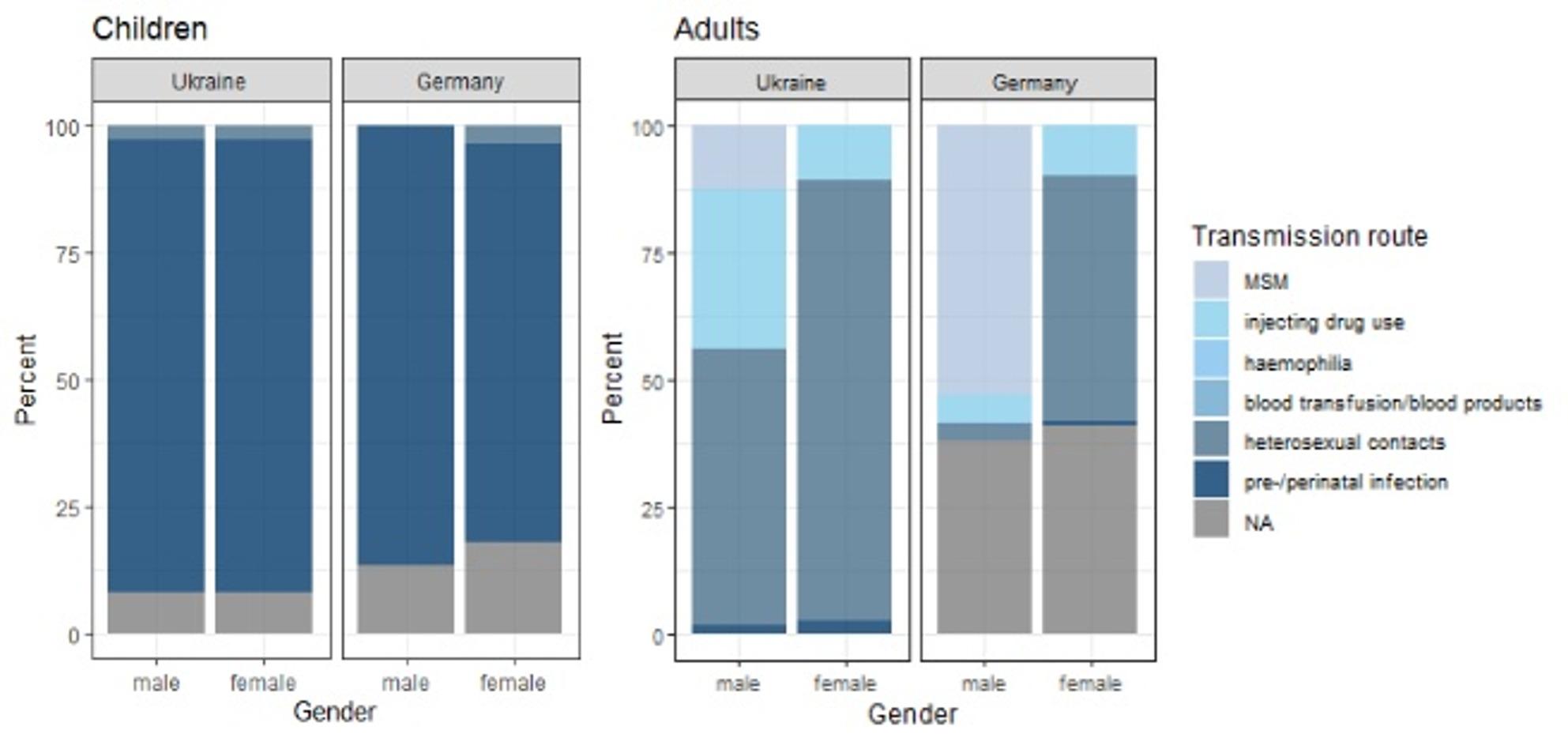
Reported transmission routes among adults and children living with HIV from UKR and GER (including unconfirmed infections), Germany, 03/2022-12/2023

Among PLHIV aged 15 years and older from UKR, heterosexual contact was the most frequently reported transmission route overall (*n* = 949; 74%), both among males (*n* = 272; 54%) and females (*n* = 677; 87%), followed by injecting drug use (IDU) (overall: 20%; males: 31%; females: 11%) (Table 1; Fig. 4). This distribution was consistent for both confirmed and unconfirmed infections: among unconfirmed infections, heterosexual contact as reported by 60% of males and 95% of females, while among confirmed infections, the corresponding proportions were 54% and 86%, respectively. Among female PLHIV from GER, heterosexual contact was also the most frequently reported transmission route, though to a lesser extent (*n* = 211; 49%), followed by IDU (*n* = 45; 10%). In contrast, the majority of male PLHIV from GER were men who have sex with men (MSM) (*n* = 1,335; 53%) which was also the most frequently reported mode of transmission among all PLHIV from GER (45%) (Table 1; Fig. 4). Among PLHIV from GER with confirmed infections, we observed a similar pattern: heterosexual contact was reported by 59% (*n* = 221) of females, and MSM by 58% (*n* = 1311) of male PLHIV. Most PLHIV from GER with unconfirmed infections lacked information on transmission route (males: *n* = 216, 86%; females: *n* = 77, 96%).

#### Children

Among children under 15 years of age, reported transmission routes were similar between females and males in both countries. Pre- and perinatal transmission was the most frequently reported route, accounting for 83% (*n* = 48) of all notifications among children from GER and 89% (*n* = 66) of those from UKR (Table 1; Fig. 4). Heterosexual contact was reported infrequently (UKR: *n* = 2, 3%; GER: *n* = 1, 2%).

Among children with unconfirmed infections, pre- and perinatal transmission was the only reported transmission route for PLHIV from both countries (UKR: *n* = 24, 100%; GER: *n* = 46, 85%), while the remaining children from GER lacked data on transmission route.

### Diagnostic statuses

Table 2 presents the categorization of PLHIV from UKR and GER according to diagnostic status, i.e. whether the HIV diagnoses was already known or probably known prior to notification. Notifier comments indicating a diagnosis made abroad and known before were unevenly distributed between countries: among PLHIV from UKR, 41% of children (*n* = 30) and 34% (*n* = 440) of adults were reported with such a comment (overall: *n* = 470; 35%). In contrast, among PLHIV from GER, only a small number (*n* = 11, < 1%) were reported with a comment suggesting a prior diagnosis abroad, all of whom were adults.

**Table 2:** Diagnostic statuses of PLHIV from UKR and GER with data on age, 03/2022-12/2023

	UKR (*n* = 1,360)		GER (*n* = 3,030)	
	Adults (*n* = 1,286)	Children (*n* = 74)	Adults (*n* = 2,972)	Children (*n* = 58)
Prior known diagnosis Notifier comment	440 (34%)	30 (41%)	11 (< 1%)	0 (0%)
Probably prior known diagnosis VL < = 200 copies/ml and/or CD4 > 500 cells/µl	243(19%)	25 (34%)	279 (9%)	18 (31%)
Viral load below detection limit	189 (78%)	16 (64%)	71 (25%)	18 (100%)
% confirmed infections	217 (89%)	10 (40%)	256 (92%)	0 (0%)
Unclear VL > 200 copies/ml and/or CD4 < = 500 cells/µl	215 (17%)	6 (8%)	1,228 (41%)	4 (7%)
% confirmed infections	205 (95%)	6 (100%)	1135 (92%)	4 (100%)
No data	388 (30%)	13 (13%)	1,454 (49%)	36 (62%)
% confirmed infections	364 (94%)	6 (46%)	1238 (85%)	0 (0%)

Proportions of probably known diagnoses among children – based on reported viral loads and CD4 cell counts – were similar between UKR (34%) and GER (31%). However, all children from GER in this category had unconfirmed infections with viral loads below the detection limit, which may indicate false positives rather than probably known diagnoses.

Overall, the combined proportion of known and probably known diagnoses was substantially higher among both adults (53%) and children (75%) from UKR compared with those from GER (9% and 31%).

A detailed analysis of viral load and CD4 cell count distributions is provided in Additional file 1.

## Discussion

### Increase in HIV notifications linked to Ukraine

As expected, we observed a steep increase in notifications concerning PLHIV from Ukraine in March and April 2022, coinciding with the arrival of the first refugees from Ukraine in Germany after the beginning of Russia’s war of aggression [16]. This aligns with previous findings from 2022 showing a tenfold increase in HIV notifications concerning PLHIV from Ukraine in the EU/EEA countries [17]. In our study, the proportion of PLHIV from Ukraine among all HIV notifications remained substantial throughout the study period until December 2023. However, the actual number of PLHIV among the migrant population from Ukraine may be higher, as in 2020 it was estimated that only 75% percent of PLHIV in Ukraine were aware of their infection [18]. Ensuring rapid integration of those already diagnosed into the German HIV care system, as well as facilitating access to testing and prevention for those unaware of their status, remains essential.

Unlike refugees from other countries, people migrating from Ukraine were granted full access to the German statutory health insurance scheme as of June 2022 and are free to choose their place of residence in Germany [19]. This creates both opportunities and challenges in terms of HIV prevention and care in Germany. Access to all health services provides a basis for an integration into regular HIV care service schemes. However, the implementation of legal regulations may not automatically translate into zero barriers to accessing health services. Prior research has shown that Ukrainian refugees often face difficulties navigating the German healthcare system due to inherent differences compared to the Ukrainian system and in obtaining information about where to best access health services [20]. Given the regional variation and complexity of testing and treatment structures in Germany – including governmental and non-governmental providers [21] – clear guidance and coordinated support are critical. Limited official support from have already led to the establishment of non-governmental initiatives specifically supporting PLHIV from Ukraine in Germany since August 2022 [22]. Strengthening systematic support mechanisms and improving coordination among stakeholders could help ensuring timely access to HIV care for PLHIV who migrated to Germany.

Language barriers represent an additional challenge. Both Ukrainian refugees and healthcare providers in Germany report difficulties during medical consultations [20]. Interpreter services usually must be organized and paid for by patients themselves [20, 23]. Given sustained migration from Ukraine and continued high notification numbers, nationwide, coordinated effort are needed to optimize HIV and general healthcare for this population.

### Differences in characteristics call for targeted strategies

Compared with PLHIV from Germany, PLHIV from Ukraine showed distinct characteristics in terms of gender and reported transmission routes. The proportion of female PLHIV was markedly higher among PLHIV from Ukraine, reflecting both the gender distribution of Ukrainian refugees in Germany – about 64% female as of July 2024 [2, 7]– and the higher proportion of women living with HIV in Ukraine (about 46% of PLHIV; male-to-female ratio of 1.6 among new infections in 2020) [24]. In contrast, only around 10% of PLHIV without migration background in Germany were female at the end of 2023 [12]. 

These gender differences also partially explain variations in transmission routes. The lower extent of MSM as transmission risk among PLHIV from Ukraine is a direct consequence of the higher proportion of female PLHIV. Yet, we found heterosexual contact to be the most frequently reported transmission route among female and male PLHIV from Ukraine. This aligns with previous findings indicating heterosexual contacts being the predominant mode of HIV transmission in Ukraine [18], although it may partially reflect underreporting of MSM or IDU transmission due to stigma [18, 25, 26]. In our study, IDU accounted for one-fifth of notifications about adult PLHIV from Ukraine but only for 6% of those from Germany. Other studies from Ukraine suggest that IDU might contribute to up to about 60% of HIV transmission [26], including indirect infections through heterosexual contacts with people who inject drugs (PWID) who are living with HIV [27]. 

Although the PLHIV population migrating to Germany is potentially not fully representative of the general PLHIV population in Ukraine, our findings indicate substantial differences in the gender distributions and modes of transmission between PLHIV from Ukraine and Germany. According to WHO recommendations, HIV care should follow people-centred, differentiated approaches tailored to the population-specific needs [9]. Germany’s national HIV strategy, while strongly focused on MSM, has set targets to improve HIV services for other key populations – including PWID, sex workers and migrants from sub-Saharan Africa by 2030 [28]. However, a recent midterm evaluation found that activities to address the needs of PLHIV beyond MSM are lagging behind [21]. The arrival of refugees from Ukraine underscores the urgency of strengthening migrant-inclusive HIV prevention, testing and treatment services in Germany – particularly targeting PWID and women. This includes the establishing harmonized guidelines for refugee health covering the provision of testing services for HIV and treatment offers.

### Mother-to-child transmission and HIV in children

We found a slightly higher proportion of HIV notifications concerning children under 15 years of age among PLHIV from Ukraine than from Germany. This reflects both demographics differences in the refugee population from Ukraine and the higher prevalence of pre- and perinatal transmission in Ukraine [2, 7]. In 2021, HIV prevalence among pregnant women in Ukraine was estimated at 0.72%, with a mother-to-child transmission (MTCT) rate of about 8.8 per 100,000 in 2021 [18]. In contrast, in Germany this figure was reported at around 0.8 per 100,000 in 2016 [29]. 

Including notifications not fulfilling the reference definition means that some reported infections among children – particularly in Germany – may represent transient maternal antibodies rather than true infections [30]. This likely explains the higher proportion of unconfirmed infections and without comments on known diagnoses among children from Germany. In contrast, the majority of children from Ukraine had confirmed infections or comments indicating known diagnoses, suggesting genuine HIV infections.

These findings call for a stronger focus on HIV prevention and care services for children and adolescents to meet the needs of PLHIV from Ukraine. Interventions might include improving awareness among pregnant women, as we found the vast majority of children linked to Ukraine to have acquired HIV infection through pre- or perinatal transmission. This is supported previous work highlighting the importance of optimal HIV care for pregnant women and children among Ukrainian refugees in Germany [31]. For adolescents, this might include education about safe sex practices, as knowledge gaps in this area reportedly persist in Ukraine [32]. 

### HIV diagnosis among PLHIV from Ukraine are mostly known and treated

As expected, we identified a high proportion of known and probably known diagnoses among PLHIV from Ukraine. Among PLHIV from Ukraine with unconfirmed infections, the higher proportion of those with data on viral loads may indicate a larger extent of already known infections, where testing was limited to viral load assessment for treatment continuation – consistent with WHO recommendations [9]. The large share of PLHIV from Ukraine with viral loads below detection limits supports this assumption.

Our findings are consistent with other EU/EEA countries` data, which showed an increase in notifications about previous positive HIV diagnoses from 8.8% in 2021 to 16.6% in 2022 [2]. The majority of PLHIV with known diagnoses had a different country of origin than the reporting country with 44.7% coming from Central and Eastern Europe [2]. In Ukraine, an estimated 83% of PLHIV aware of their infection were receiving antiretroviral therapy (ART) in 2021 [18]. Early research from Germany corroborates this: studies conducted at infectious disease outpatient clinics in Hamburg and a paediatric unit in Berlin reported nearly all treated PLHIV from Ukraine in 2022 were aware of their infection and receiving ART before arrival [31, 33]. 

Ensuring rapid treatment continuation and regimen adaptation for PLHIV migrating from Ukraine is therefore critical. The predominant the single-tablet regimens in Ukrainian treatment schemes are not licensed in the EU and must be replaced [33, 34]. 

### Importance of high-quality surveillance data

Our findings must be viewed in the light of several sources of uncertainty: Missing information on country of origin or infection likely led to underestimation of the actual number of PLHIV from Ukraine. Moreover, incomplete data on known diagnoses, CD4 cell counts, and viral loads limited our ability to establish diagnostic statuses comprehensively. Nevertheless, the somewhat lower proportion of missing data among PLHIV from Ukraine supports the credibility of our findings and may reflect targeted efforts by the RKI to improve reporting completeness for notifications concerning PLHIV from Ukraine in 2022 [11]. Germany’s reference definition for HIV cases (here described as ‘confirmed infections’), which is based on the first diagnosis within the country, complicates the differentiation between new and previously known diagnoses among migrant populations [8]. Including unconfirmed infections may also lead to overestimation due to false positives, as seen among children from Germany in this analysis. Despite these limitations, our analysis demonstrates the potential of routine surveillance data as a resource-efficient tool to provide evidence about PLHIV among migrants and inform public health strategies. To enhance this potential, systematic improvements in data quality – particularly complete reporting of country of infection and origin, and diagnostic history might be useful. The European Centre for Disease Prevention and Control (ECDC) has launched efforts to standardize data collection on previous positive diagnoses across Europe, representing a step forward [2]. 

### Limitations

Our findings are subject to several limitations. Estimates of PLHIV from Ukraine are likely conservative due to undiagnosed infections and potential underreporting. Therefore, we acknowledge that we can only estimate the extent of known diagnoses among the notified infections, but not among all existing HIV infections among migrants from Ukraine. The use of CD4 cell counts and viral loads as proxies for diagnostic status may also misclassify some recent infections as known diagnoses. Including unconfirmed infections might further overestimate the number of PLHIV due to potential false positives, particularly among children from Germany. Additionally, the amount of missing data in general reduces the credibility of our findings. Despite these constraints, the high proportion of comments on known diagnoses among PLHIV from Ukraine strengthens the validity of our findings and provide insights for public health policy and a basis for further research.

## Conclusions

The marked rise in the number of PLHIV from Ukraine since 2022 highlights the need for strategies to provide them with rapidly accessible HIV prevention, testing and care structures in Germany. Following people-centred approaches, differences in demographic and clinical characteristics among PLHIV from Ukraine must inform tailored measures, especially for women and children. Germany’s national HIV strategy, currently largely focussed on MSM, should be broadened to address the needs of migrant populations. Our findings point towards a high proportion of known and treated HIV infections among PLHIV from Ukraine, which underscores the importance of ensuring rapid treatment continuation and optimal adaptation of ART schemes to the German context. Finally, this analysis illustrates the potential of routine surveillance data to inform migrant-inclusive HIV policies and the necessity of further improvements in data quality.

## Supplementary Information

Supplementary Material 1

## Data Availability

The datasets used and/or analysed during the current study are available from the corresponding author on reasonable request.

## Abbreviations

ART: Antiretroviral therapy
ECDC: European Centre for Disease Prevention and Control
EU/EEA: European Union/European Economic Area
GER: Germany
HIV: Human Immunodeficiency Virus
IDU: Injecting drug use
IfSG: Infection Protection Act
MSM: Men who have sex with men
MTCT: Mother to child transmission
PLHIV: People living with HIV
PrEP: Pre-exposure prophylaxis
PWID: People who inject drugs
RKI: Robert Koch-Institute
UKR: Ukraine
WHO: World Health Organization
