# Supplementary material for: The potential of routine surveillance data for identifying the needs of people living with HIV among migrants: Description of German HIV notifications in the context of the Ukrainian refugee reception, 2022–2023
Source: BMC Public Health. 2026 Feb 28;26:1118. doi: 10.1186/s12889-026-26787-6 (PMC13059271; doi:10.1186/s12889-026-26787-6)

## Additional file 1

#### **Viral load**

**Data completeness**

For 789 (58%) PLHIV from UKR data on viral load was reported, compared to 1,475 (48%) PLHIV from GER (Table 1). In the first group, this percentage was higher among PLHIV with unconfirmed infections (n=70, 65%), whereas in the second group it was lower (n=126, 33%). Similarly, PLHIV from UKR which were reported with a notifier comment on a diagnosis abroad were more often reported with viral loads (n=354, 71%) than PLHIV from GER (n=6, 55%).

Among PLHIV reported without a comment on a diagnosis abroad, percentages with reported viral loads where similar between both groups (UKR: n=435 (50%); GER: n=1,469 (49%)), but higher for children (n=29, 66%) than adults (n=427, 51%) from UKR, whereas among PLHIV from GER the percentage of notifications with reported viral loads was lower among children (n=22, 38%) than adults (n=1444, 49%).

Percentages of notifications with reports on viral loads were similar between confirmed (50%; n=408) and unconfirmed infections (59%, n=48) among PLHIV with UKR without a notifier comment on a diagnosis abroad. Regarding PLHIV from GER without such a comment, the percentage who were reported with viral loads was higher (51%; n=1343) among PLHIV with confirmed infections than among those with unconfirmed infections (33%; n=126).

**Reported viral load among PLHIV without notifier comment on prior diagnosis**

Among PLHIV without a notifier comment on a diagnosis abroad and data on viral load, those from GER had higher reported viral loads (mean: 1,015,459 copies/ml; median: 106,000 copies/ml) than those from UKR (mean: 466,467 copies/ml; median: 456 copies/ml) (Figure 5).

Among adults from UKR with unknown diagnostic status, but information on viral loads, 45% (n=205) had a reported viral load below detection limit and 49% (n=224) up to 200 copies/ml compared to 6% (n=90) and 8% (n=110) respectively among PLHIV from GER. Among children from UKR without notifier comments about a prior diagnosis and information on viral loads, 55% (n=16) had a reported viral load below detection limit, of which 3 had confirmed infections (19%). Among children from GER without a notifier comment but data on viral loads, 82% (n=18) had a reported viral load below detection limit, of which all had unconfirmed infections.

**Figure 5.** Viral loads among PLHIV from UKR and GER without notifier comment on diagnosis abroad, 03/2022-12/2023.


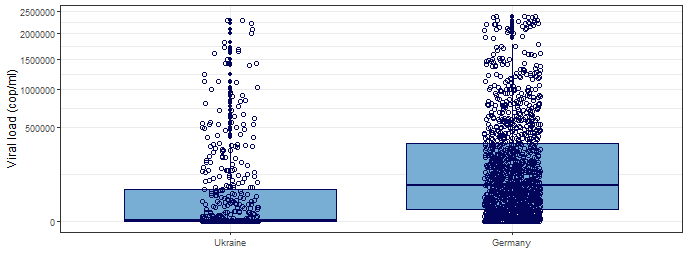


#### **CD4 cell count**

**Data completeness**

For 647 (48%) PLHIV from UKR data on CD4 cell counts was available as compared to for 861 (28%) PLHIV from GER Germany (Table 1). In the first group, this percentage was higher among PLHIV with unconfirmed infections (n=60, 56%), whereas in the second group it was lower (n=41, 11%).

In both groups a higher percentage of data on CD4 cell counts was reported among those with a comment on a diagnosis abroad (UKR: n=294 (62%); GER: n=7 (64%). Among those without a comment, percentages of reports on CD4 cell counts were lower (UKR: n=353 (40%); GER: n=854 (28%)) but similar between adults (UKR: n=327 (39%); GER: n=840 (28%)) and children (UKR: n=26 (46%); GER: n=14, (23%)) in both groups.

Percentages with available data on CD4 cell counts were comparable between PLHIV from UKR without a notifier comment about a prior diagnosis with confirmed (39%; n=314) and unconfirmed infections (48%; n=39), whereas more PLHIV from GER with confirmed infections (31%; n=813) had data on CD4 cell counts than those with unconfirmed infections (11%; n=41).

**Reported CD4 cell counts among PLHIV with unspecified diagnostic status**

Of PLHIV reported without a comment on a diagnosis abroad, but information on CD4 cell counts, a higher proportion had a reported CD4 cell count above 500/μl among PLHIV from UKR (n=165, 47%) than from GER (n=230, 27%), whereas the proportion with a CD4 cell count below 200/μl was higher among the latter (n=306, 36%) than the former (n=88, 25%) (Figure 6). Reported CD4 cell counts were higher among children than among adults for both PLHIV from UKR (>500/μl: n=17 (90%) vs. n=148 (44%)) and GER (>500/μl: n=13 (93%) vs. n=217 (26%)).

For both countries, CD4 cell counts were higher among PLHIV with unconfirmed infection (>500/μl: UKR: 64%; n=25; GER: 56%; n=23) than among those with confirmed infection (>500/μl: UKR: 45%; n=140; GER: 26%; n=207).

**Figure 6.** Reported CD4 cell counts of PLHIV from GER and UKR without notifier comment on prior diagnosis, 03/2022-12/2023.


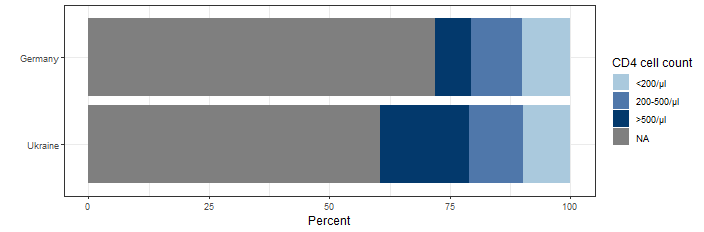

Supplement: Supplementary file 1 — Supplementary Material 1 [file 12889_2026_26787_MOESM1_ESM.docx]
